# How and why SGLT2 inhibitors should be explored as potential treatment option in diabetic retinopathy: clinical concept and methodology

**DOI:** 10.1177/2042018819891886

**Published:** 2019-12-11

**Authors:** Marcus May, Theodor Framke, Bernd Junker, Carsten Framme, Amelie Pielen*, Christoph Schindler*

**Affiliations:** Hannover Medical School, MHH CRC Core Facility, Feodor-Lynen-Strasse 15, Hannover, 30625, Germany; Institute of Biostatistics, Hannover Medical School, Hannover, Germany; University Eye Hospital, Hannover, Germany; University Eye Hospital, Hannover, Germany; University Eye Hospital, Hannover, Germany; MHH Clinical Research Center Core Facility (OE 8660) and Center for Pharmacology and Toxicology, Hannover, Germany

**Keywords:** academia, diabetes mellitus, diabetic macula edema, diabetic retinopathy, methods, pharmacology, SGLT2 Inhibition, trial design

## Abstract

Patients suffering from type 2 diabetes are at an increased risk of developing classical microvascular complications such as retinopathy, neuropathy, and nephropathy, which represent a significant health burden. Tight control of blood glucose, blood pressure, and serum cholesterol reduce the risk of microvascular complications but effective pharmacologically targeted treatment options for the treatment and prevention of diabetic microangiopathy are still lacking. Pharmacological inhibition of sodium glucose cotransporter 2 (SGLT2) might have the potential to directly protect against microvascular complications and could represent a potential treatment option. Randomized controlled clinical proof of concept trials are needed to investigate a potential central role of SGLT2 inhibitors in the prevention of diabetic microangiopathy and its classical clinical complications of retinopathy, neuropathy, and nephropathy.

## Introduction

Overall life expectancy is increasing worldwide and the majority of the population is experiencing continuous weight gain.^1^ This prevailing development results in a dramatic increase in the global incidence of diabetes mellitus.^2^ Although type 1 diabetes is characterized by insulin deficiency caused by autoimmune beta cell destruction in the endocrine pancreas, type 2 diabetes is characterized by insulin resistance with high insulin levels in its early stages which results in impaired insulin production in later stages. In both diseases, elevated glucose levels, either owing to insulin resistance or insulin deficiency, are associated with adverse health problems making diabetes mellitus the ninth major cause of reduced life expectancy worldwide.^2^ Microvascular diseases such as nephropathy, retinopathy, and neuropathy and macrovascular diseases such as heart disease, stroke, and peripheral artery disease are typical complications that are causative for the increased mortality. Type 2 diabetic patients are twice as likely to develop cardiovascular diseases compared with people without diabetes and their risk of death from vascular causes is doubled.^2^ With a prevalence of about 50%, microvascular complications are even more common in diabetic patients and thus present a huge health burden.^2^ Chronic hyperglycemia and the corresponding glucotoxicity induce and enhance inflammation and progress microangiopathy that results, among other conditions, in diabetic retinopathy (DR), microvascular damage of the retina. DR presents a leading cause of visual loss globally.^3^ The prevalence is very high, up to one out of three diabetic patients has detectable DR.^2^ Moreover, there is a strong relation between DR and other microangiopathies^3^ and even a correlation to macrovascular damages.^4^ The retina is a microvascular bed that can be observed directly and repeatedly. Thus, DR as a disease pattern is a promising marker to study the pathogenesis and natural history of diabetic microangiopathy as well as the effects of potential therapeutic treatments.

To date, there are still no targeted treatments available to prevent the progression of DR in the early stages. Diabetic macular edema may occur in patients with nonproliferative and proliferative DR and is the major cause for visual impairment in patients with diabetes.^3^ Intravitreal anti-vascular endothelial growth factor (anti-VEGF) drugs and intravitreal corticosteroids are well-tolerated and effective treatment options in patients with diabetic macular edema.^5^ Laser photocoagulation of the peripheral retina is used to treat proliferative DR and prevent (further) loss of vision, vitreoretinal surgery is needed if vitreous hemorrhages or tractive retinal detachment occur. Often these late proliferative DR stages are accompanied by permanent reduced visual acuity. The available ophthalmological treatment options are predominantly focused on the end stage of the disease and do not address the early and potentially reversible microvascular changes leading to DR. New targeted therapies are urgently required to prevent or slow down the progression of DR.^6^

In the following sections we give a short overview on the pathophysiology of DR, explain potential treatment options, and delineate why we think that sodium glucose cotransporter 2 (SGLT2) inhibitors might be a valuable treatment option and should further be investigated. Finally, we report our recent experiences in conducting such a clinical trial as a monocenter approach and share our knowledge on what might be a promising trial design for future investigations of SGLT2 inhibitors in DR.

## Pathophysiology of DR and potential treatment options

**Figure 1.**
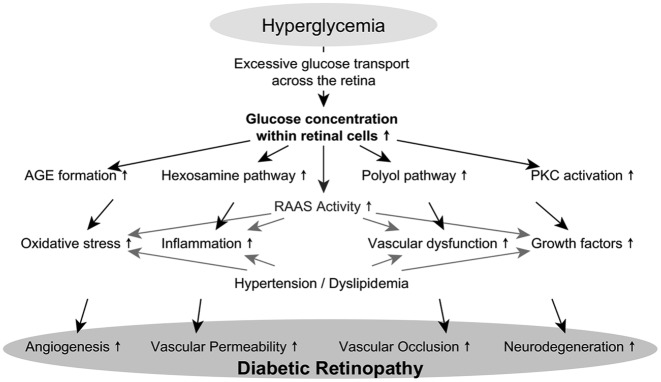
Pathomechanisms involved in the pathogenesis of diabetic retinopathy. Intracellular hyperglycemia induces the increased formation of advanced glycation end products (AGEs), upregulated hexosamine pathway, increased flux through the polyol pathway, and increased activation of protein kinase C (PKC). Because of these toxic pathways, reactive oxygen species and thus oxidative stress is increased, expression of inflammatory proteins and growth factors are triggered and, ultimately, damage to the retinal microvasculature is induced. These cell toxic consequences are worsened by activation of the renin–angiotensin–aldosterone system (RAAS) in retinal cells, uncontrolled hypertension, and dyslipidemia. Increased angiogenesis, vascular permeability, vascular occlusion, and neurodegeneration present the pathologic correlate and together characterize the pathology of diabetic retinopathy.

**Figure 2.**
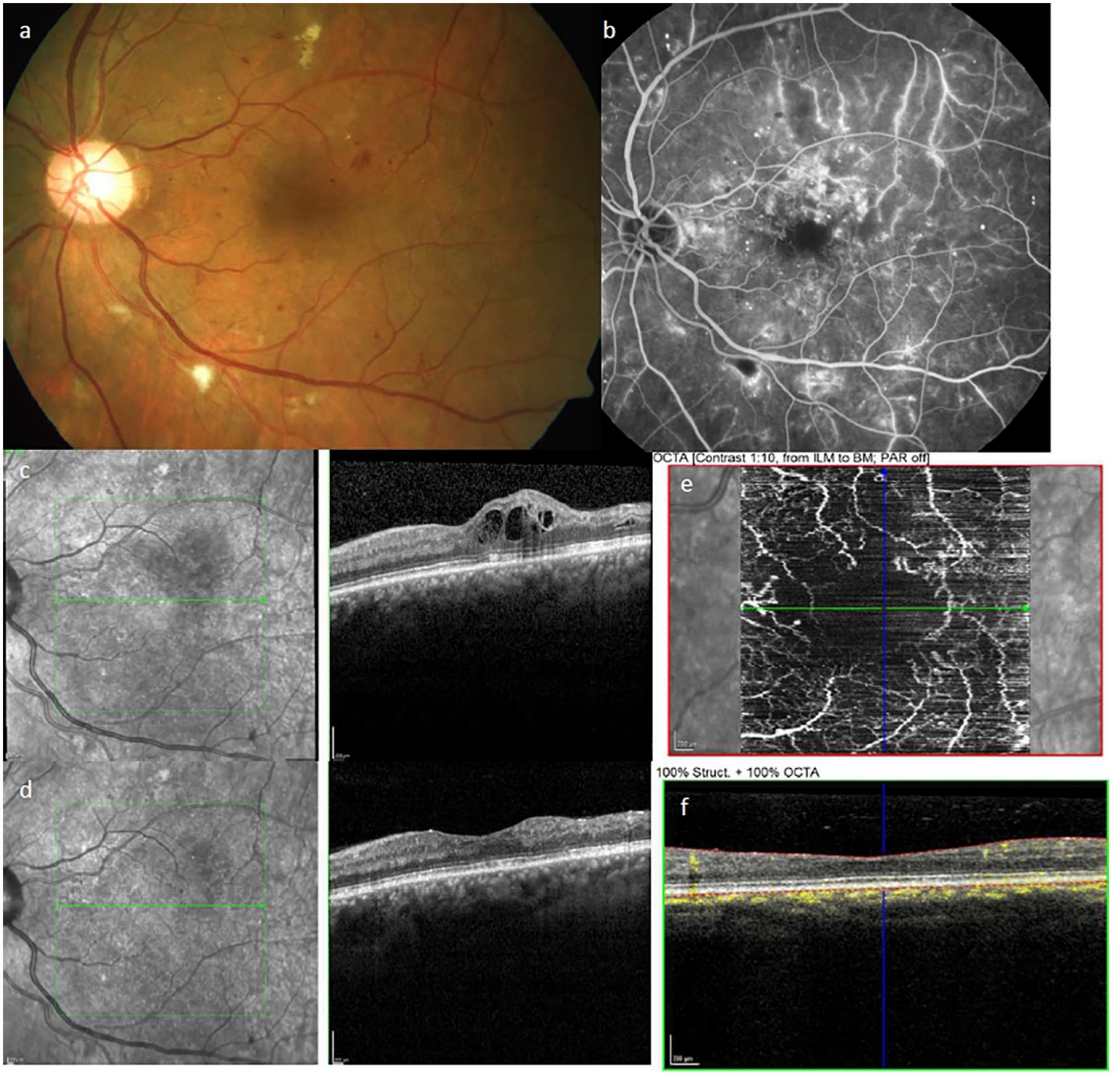
Ophthalmologic findings in patients with diabetes. Examples of pathological ophthalmologic findings in the left eye of a patient with diabetes. (a) Fundus photography showing optic nerve head and macula with signs of moderate DR: microaneurysms (red dots), small hemorrhages (red spots), hard exudates (yellow spots), and cotton-wool spots (white spots). (b) Fluorescein angiography showing the dye within the retinal vessels and capillaries, leakage of the dye outside the vessel is visible in multiple spots. (c) Spectral-domain OCT showing diabetic macula edema, the intraretinal fluid appears as black circles. (d) OCT showing near normal retinal thickness after intravitreal injection of anti-VEGF, the intraretinal fluid is almost completely resorbed. (e) OCT-angiography (without dye) showing the flow within the retinal microvessels around the central avascular arcade and (f) the corresponding OCT-angiography scan highlighting the particular segmentation of the retina (red lines) and the flow measurement (yellow) within all layers of the retina.

The pathophysiology of DR has been extensively studied. The underlying mechanisms are complex and despite the considerable amount of scientific research in this field, several unanswered questions remain.^7^ This manuscript reflects on some potential targeted treatment options and necessary clinical research concepts. Describing all involved pathomechanisms in detail is beyond its scope and current reviews delineating this topic in detail are already available.^6–8^ In brief, there are multiple contributing biochemical pathways including the polyol pathway, hexosamine pathway, protein kinase C (PKC) activation, and advanced glycation end product (AGE) formation which lead to pathological microvascular alterations and ultimately to DR.^9^ In Figure 1, the pathogenesis of DR is delineated. Intracellular hyperglycemia induces the increased formation of AGEs, increases the hexosamine pathway and flux through the polyol pathway, and results in PKC activation. Further on, antioxidant capacity is reduced by activation of the polyol pathway and thus retinal cells are exposed to increased oxidative stress. Increased availability of AGEs and PKC activation alter the expression of cytokines, growth factors, endothelial nitric oxide synthase, coagulation factors, transcription factors, and reactive oxygen species. Finally, induction of the hexosamine pathway leads to UDP-N-acetylglucosamine production, which also alters gene expression. Together these pathways, their downstream processes specifically, lead to vascular dysfunction, inflammation, oxidative stress, and altered gene expression. These processes are even accelerated by activation of the retinal renin–angiotensin–aldosterone system, uncontrolled hypertension, and dyslipidemia. Resulting degeneration of neuronal cells of the retina, angiogenesis, and vascular dysfunction finally lead to tissue damage and clinically imposing loss of vision.^8,10^ Reduced vision is the main symptom of DR but only occurs when the condition is advanced and affects either the center of the retina (diabetic macular edema or ischemic maculopathy) or so much of the peripheral retina in proliferative stages that vitreous hemorrhages or retinal detachment occur. Unfortunately, during the nonproliferative DR stage patients usually have no symptoms and normal vision, leaving patients often unaware of the condition. On routine ophthalmological examination, retinal changes may be very discrete in nonproliferative DR. Even in this stage however, microaneurysms (MAs), pericyte ghosts, acellular capillaries, thickening of the capillary basement membrane, hemorrhages, and hard exudates can be present, with MAs being the earliest ophthalmoscopically detectable clinical manifestation of DR.^9^ Characteristic features of DR detectable by ophthalmoscopic examination are hemorrhages, MAs and microvascular abnormalities such as dilated capillaries, cotton wool spots (round or oval spots with feathered edges, which represent local ischemia of the neuroretina), hard exudates (lipid deposits), retinal edema, and intraretinal neovascularization (example given in Figure 2).^9^ The Early Treatment of Diabetic Retinopathy Study (ETDRS) examined fundus photographic risk factors for progression of DR over 5 years and established a 13-level scale to describe DR severity and change of severity over time.^11,12^ When nonproliferative DR progresses to the next stage, it is called proliferative DR. The pathognomonic for this advanced stage is the growth of extraretinal new blood vessels from preexisting vessels (neoangiogenesis) and formation of fibrovascular scar tissue. Patients may experience sight threatening vitreous hemorrhage or retinal detachment due to traction. As a result, patients invariably experience vision loss at this advanced stage. Increasing hypoxia due to vascular occlusion induces VEGF production which plays a major pathophysiological role in proliferative DR and macular edema.^6^

In the early stages of diabetes, DR progression can be effectively slowed down by both tight blood glucose and blood pressure control.^13,14^ Up to now, optimized metabolic control in the early stages of DR seems to be the most effective treatment with no specific treatment option available for early ophthalmological changes.^6^ Repeated intravitreal injections of VEGF inhibitors show a marked efficiency in patients with severe disease.^5^ Anti-VEGF treatment does not only reduce macular edema and improve vision, it also shows an improvement in the extent of DR-induced vascular changes.^15^ Anti-VEGF intravitreal injections however, are needed every month at the start of treatment and should be given repeatedly over many months. Data from trials leading to the approval of ranibizumab and aflibercept for diabetic macular edema as well as real-world data show that best visual acuity is achieved with a mean of 7–8 injections in the first year, 4–6 injections in the second year and less than 3 in the third year.^16^ Effective treatment regimens are a great burden for patients and supporting families. It would be best to treat DR before diabetic macular edema evolves to prevent vision loss. A treatment option for early stages of DR is urgently needed to improve patient health burden and quality of life.^8^

The retina with its high metabolic activity is dependent on the systemic circulation for the delivery of glucose.^9^ As mentioned previously, the common cascade leading to the pathogenesis of DR is excessive transport of glucose across the retina, high glucose concentrations within cells of the retina and, thus, induction of cell toxic pathways.^8^ Glucose transporter and sodium-dependent glucose cotransporter (SGLT) regulate glucose entry into the retinal cells. Two transporters, SGLT1 and SGLT2, have been particularly well characterized. SGLT1 plays a major role in absorbing glucose from the lumen of the intestine whereas SGLT2 is mainly expressed in the renal proximal tubules and is required for the reabsorption of glucose. Most notably, SGLT1 and SGLT2 are also expressed in the eye and the retina.^9^

In the last couple of years, a new class of antidiabetic drugs is available which inhibits SGLT2 and thereby decreases reabsorption of glucose from the renal proximal tubules, thereby increasing renal glucose excretion. With these drugs, blood glucose is effectively lowered and metabolic and hemodynamic risk factors like blood pressure and body weight, which are tightly linked to diabetic microangiopathy, are effectively ameliorated.^17,18^ Lowering blood pressure and body weight clearly contributes to the observed benefits of cardiovascular risk reduction and slower progression of renal disease.^19^ Regardless of the cause, the observed results from cardiovascular endpoint trials are very impressive and, presumably, treatment guidelines for patients with diabetes will soon be amended accordingly.

## Scientific rationale why especially SGLT2 inhibitors might be an effective pharmacologic treatment for prevention and treatment of DR

Conventional antidiabetic therapies, such as sulfonylureas-based therapy regimens, act pharmacologically by enhancing insulin secretion.^20^ Efficacy is limited because of progressing β-cell dysfunction and desensitization of insulin signaling resulting in increased peripheral insulin resistance. On the other hand, increased insulin resistance and elevated insulin levels are associated with adverse macrovascular and microvascular consequences.^2^ Thus, a more rational approach in these patients may be the reduction of glucotoxicity, insulin resistance, and hyperinsulinemia by adding a SGLT2 inhibitor.^9^

SGLT2 inhibitors are a promising new drug class for the treatment of type 2 diabetes reducing blood glucose levels in type 2 diabetes patients by inhibiting glucose reabsorption in the proximal tubule, subsequently increasing renal glucose excretion. Approved agents for the treatment of type 2 diabetes are empagliflozin (Boehringer Ingelheim, Germany), dapagliflozin (AstraZeneca, UK), and canagliflozin (Johnson & Johnson, US, no longer available in Germany). Pharmacokinetics are very similar for all SGLT2 inhibitors and these drugs have been shown to be safe and well tolerated. With the exception of canagliflozin, which should be given in a higher daily dose when co-administered with CYP450 inducers such as rifampicin, phenytoin, or ritonavir, SGLT2 inhibitors do not show any clinically relevant interactions.^20^ Treatment with empagliflozin, dapagliflozin, or canagliflozin reduces cardiovascular mortality in patients with type 2 diabetes at high-risk for cardiovascular events when added to the standard of care.^19^ Moreover, diabetic microangiopathy complications such as diabetic nephropathy are significantly improved compared with a placebo when patients are treated with one of the three agents.^21^ Whether only these three specific SGLT2 inhibitors are beneficial in patients with diabetes mellitus with regard to microangiopathy is currently unknown but available meta-analyses support the concept of a class effect.^19,21^

By addressing its fundamental disease causes SGLT2 inhibitors may be particularly suitable also in improving DR through substantial improvement of systemic glucose metabolism, lowering of blood pressure, and reduction of body weight.^19^ SGLT2 inhibitors enhance glycosuria and lead to a reduction in insulin secretion, improved beta cell function, lower tissue glucose uptake, and improved insulin sensitivity.^22^ Moreover, SGLT2 inhibitors ameliorate metabolic and hemodynamic risk factors tightly linked with DR, such as blood pressure and body weight.^17,18^ The reduction of sympathetic vasomotor tone, and renin–angiotensin system activity might provide additive benefits in treating DR.^8,9^ SGLT2 inhibitors remove excessive glucose from the retinal microcirculation and hence reduce glucotoxicity, oxidative stress, low-grade inflammation, and restore insulin signaling. By preventing continued glucose-induced vascular dysfunction and endothelial dysfunction, progression of microangiopathy and especially DR are improved.^23^ Expression of SGLT1 and SGLT2 has been reported in the eye and the retina and, in line with this finding, positive effects on the eye and retina have been described in rats treated with SGLT2 inhibitors.^9^ *Post hoc* analysis of a subgroup of patients with DR from the EMPA-REG OUTCOME trial with high-risk for progression showed no treatment associated risk for the development or worsening of DR.^24^ In this trial, DR was not assessed regularly in the trial participants and reported only in case of adverse events. Nevertheless, there was a lower number of patients with retinal events in the placebo group showing an insignificant trend to risk reduction with empagliflozin (HR 0.78, *p* = 0.17).^24^ In another clinical trial Ott and colleagues showed numerous beneficial effects of dapagliflozin treatment on vascular remodeling with a crossover study design and meticulous evaluation of vascular outcomes after only 6 weeks of treatment.^25^ Retinal microvasculature showed lowered retinal capillary flow and prevented retinal arteriole changes when compared with placebo. The placebo group on the other hand showed increased wall-to-lumen ratio indicative of retinal vascular hypertrophy, which was not observed in the dapagliflozin group.^25^ Moreover, Dziuba and colleagues showed that SGLT2 inhibitor treatment lowers microvascular complications in patients with early stage type 2 diabetes and postulated that only 15 patients with diabetes would be needed to show a treatment benefit in one patient (number needed to treat = 15). The analysis was performed using the Archimedes model to simulate a 20-year clinical study based on available clinical data.^26^ Available clinical evidence has shown a statistically insignificant beneficial effect. Up to now no prospective, randomized, and controlled clinical study has demonstrated the ‘beyond blood glucose control’ effect of SGLT2 inhibitors on DR because none of the studies systematically assessed the retinal pathology and progression of DR in patients with diabetes in detail before and after treatment with an SGLT2 inhibitor. Nevertheless, pharmacologic properties and the delineated observations of clinical trials suggest that SGLT2 inhibitors may possess direct beneficial properties in the prevention of DR. Therefore, clinical trials are needed with sophisticated methods to assess DR systematically.

## Suited scientific methods and biomarkers to be used in early phase proof of concept clinical trials to investigate the influence of a pharmacologic treatment on progression of DR

Staging of DR can be done using the grading guidelines established by the ETDRS group that are considered to be the gold standard in clinical trials.^11,12,27^ In short, the scale describes retinal changes from none (level 10), over nonproliferative changes mild (level 20, MAs only and level 35, hard exudates and vascular abnormalities), moderate (level 43, 47), severe (53A–D) and very severe (53E) to proliferative stages mild (61), moderate (65), high-risk (71, 75) and advanced (81, 85). Details can be found in ETDRS report No. 12.^12^ Early stages of DR are characterized by MAs, small hemorrhages, and indirect signs of vascular hyperpermeability such as hard exudates (Figure 2). Clinical examples are displayed in Figure 2. In fact, MAs are the earliest ophthalmoscopically detectable clinical manifestation of DR. Grading is done on fundus photography of the retina by blinded graders, who can be supported by automated software solutions. Different areas of special interest such as macula, optic nerve head and the periphery of the retina are analyzed and assessed in predefined fields. Computer-assisted evaluation of MA formation rate (e.g. Retmarker™, www.retmarker.com) presents a favorable tool to detect patients at risk of developing macula edema and progressive visual loss and is supported by the European Medicines Agency as a clinical endpoint.^28^ Moreover, MA formation rate is thought to be a favorable surrogate parameter and might function as a biomarker for DR progression rate with good discriminatory power requiring feasible sample sizes.^29,30^ In addition to the ETDRS severity scale, a composite clinical outcome evaluating progression to proliferative DR is useful to monitor progression based on photographic changes, angiography, plus clinically important events defining proliferative DR.^31^ Ophthalmological standard examination includes fluorescein angiography to examine perfusion status and ischemia of choroidal and retinal vessels (Figure 2). The downside of this method is the invasive procedure (intravenous dye, risk of anaphylaxy) and the long examination time (minimum of 20 minutes). The benefits of the spectral-domain optical coherence tomography (OCT) are high-resolution anatomical images of the neuroretina comparable with histological images within a very short acquisition time (seconds) (Figure 2). It is the current standard for the diagnosis of macular edema and degeneration of the optic nerve head as well as essential for the follow-up and monitoring of therapeutic effects during intravitreal treatment of macular edema.^5^ The very recent improvement in OCT technology enables OCT-angiography (OCT-A), a technique to detect and show the flow within the retinal vessels, including the microvascular capillaries without the need for a dye (Figure 2).^32^

Ophthalmological examination within a clinical trial investigating potential treatment of DR should also comprise best corrected visual acuity testing (ETDRS letters), slit lamp exam of cornea, anterior chamber and lens, fundus examination, and tonometry.

## Respective trial design to be recommended for testing SGLT2 inhibitors on DR in an early proof of concept study

**Figure 3.**
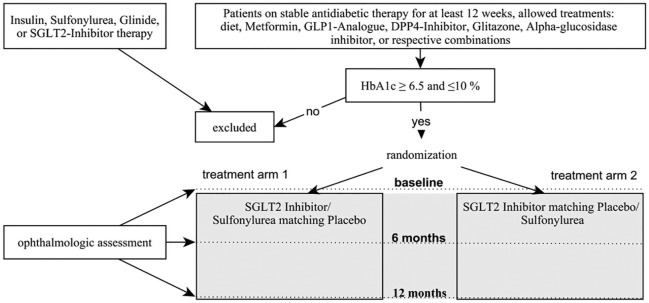
Proposed study design.

As mentioned previously, some clinical trials have already shown the potential beneficial effects of SGLT2 inhibitor treatment on retinal microcirculation and progression of DR.^24,25^ Even though a computer-based approach modeling the effects of SGLT2 inhibitors on microvascular outcomes, as conducted by Dziuba and colleagues, has already provided the first evidence supporting the hypothesis of the potential beneficial treatment effects of these drugs,^26^ this simulation study did not provide sufficient evidence to estimate the treatment effect size and proposed treatment efficiency. To reach this goal, a prospective, randomized, multicenter, double-blind, clinical proof of concept trial needs to be performed comparing SGLT2 inhibitor treatment with standard treatment specifically investigating the effects on DR. As sulfonylureas are still recommended as second-line treatment for patients who do not achieve sufficient glycemic control with metformin alone or with contraindications for metformin,^33,34^ sulfonylureas can still be regarded to be a suitable comparator in such a controlled study setting for DR. The UKPDS study revealed a clear reduction of microvascular complications in the sulfonylurea group. Intensive blood-glucose control with sulphonylureas or insulin compared with conventional treatment and risk of complications in patients with type 2 diabetes (UKPDS 33). In addition, the ongoing discussion about cardiovascular safety of sulfonylureas since the 1960s, the CAROLINA study (Cardiovascular Outcome Study of linagliptin *versus* glimepiride in type 2 diabetes) showed comparable cardiovascular safety of linagliptin and glimepiride in patients with type 2 diabetes over 6.2 years.^35^ Whereas sulfonylureas would increase insulin resistance in the retinal microvasculature, SGLT2 inhibitors are regarded as neutral which might provide additional benefit. As a suggested primary outcome, MA formation rate seems to be the best available clinical parameter to monitor early changes in DR and,^29^ therefore, seems to be an ideal primary study endpoint for a clinical proof of concept trial examining potential treatment effects in DR. Other important secondary endpoints should be DR stage (ETDRS letters),^12^ MA count, retinal thickness measured by OCT, retinal perfusion of microvasculature within the retina measured by OCT-A, intraocular lipid content (hard exudates), best corrected visual acuity (ETDRS letters), body weight and body fat mass (e.g. assessed with air displacement plethysmography or bioelectrical impedance analysis), ambulatory blood pressure, HbA1c, fasting glucose, and blood lipids. Special attention should be given to the urine status of patients: its glucose determination can unblind investigators so that appropriate steps need to be taken at the planning stage to avoid accidental unblinding. Moreover, with sulfonylurea as a comparator, the increased risk for hypoglycemia has to be accounted for, especially if patients are included with HbA1c values lower than 7% to facilitate recruitment. In this case a close safety monitoring of the patients is necessary with safety visits every 2 weeks and additional physician availability by phone recommended. Hyperglycemic and presumably hypoglycemic episodes can influence microvascular outcome.^36^ A proposed visit schedule with suggested study-related assessments for the systematic evaluation of endpoints and safety parameters is shown in Table 1. For reasons of practicability in recruitment, background standard diabetes medication should be allowed, except already ongoing treatment with an SGLT2 inhibitor or a sulfonylurea. Nevertheless, all eligible patients should be on stable antidiabetic treatment for at least 30 days before study entry as reflected by a stable HbA1c value. Suitable patients should be randomized to a 12-month double-blind treatment period with either SGLT2 inhibitor or comparator (sulfonylurea) in addition to unblinded pretreatment antidiabetic medication. A 1:1 randomization is recommended to be performed centrally and stratified for center and ETDRS level (20, mild DR, only MAs present *versus* 35, moderate DR, MAs, small hemorrhages or hard exudates present).^12^ Before and after 52 weeks of treatment, DR progression rate and the previously mentioned other clinical parameters should be assessed. The proposed study design is shown in Figure 3. A double-blind trial with SGLT2 inhibitor and sulfonylurea-based treatments requires increased efforts to preserve blindness: A double-placebo double-dummy approach for SGLT2 inhibitor and sulfonylurea tablets needs to be established.

**Table 1.** Visit schedule and study-related assessments.

Visit	Baseline visit (Screening) Ophthalmologic assessment before start of IMP treatment	Safety visits 14 days after start of IMP treatment, then every 5 weeks ± 1 week	Ophthalmologic assessment week 27 and 52 ± 1 week
Informed consent & medical eligibility review	✓		
Demographic data	✓		
General medical history and baseline conditions	✓		
Concomitant medications	✓	✓	✓
Physical examination	✓	✓	✓
Inclusion / exclusion criteria	✓		
Height and weight, waist circumference	✓		✓
Office blood pressure	✓		✓
Ambulatory blood pressure	✓		✓
12-lead ECG	✓		✓
SAE / AE	✓	✓	✓
Randomization	✓		
Study drug dispensation, accountability	✓	✓	✓
Ophthalmologic examination	✓		✓
Safety laboratory	✓	✓	✓

AE, adverse event; ECG, electrocardiogram; IMP, Investigational Medicinal Product; SAE, serious adverse event.

MA formation rate as a biomarker is sensitive enough to detect even small changes in DR progression rate after 12 months of treatment.^29^ The following subsection provides considerations for sample size, power, expected treatment effect size, and primary statistical analysis in a proof of concept trial. With the MA formation rate over 12 months as the primary endpoint, sample size considerations, and the analysis method based on count data are needed. MA formation rate for the control group can be estimated as an average (weighted by group sizes) of stratified results reported in a prospective study with similar inclusion criteria and *n* = 348 patients.^37^ Accordingly, a MA formation rate of 2.78 ± 4.04 (mean ± SD) is assumed for the control group in the power estimation. Moreover, we expect an equal standard deviation in both treatment groups. In this case, a difference in MA formation rates of 1.73 between the two treatment groups (2.78 sulfonylurea *versus* 1.05 SGLT2 inhibitor, rate ratio 0.38) can be detected with a power of 80% and a two-sided significance level of 5% based on calculations according to Tang.^38^ For the analysis of the primary endpoint, a negative binomial regression model with covariables treatment group (SGLT2 inhibitor *versus* sulfonylurea), center and ETDRS at baseline [20 (mild DR) *versus* 35 (moderate DR)] is recommended for the primary analysis. Superiority of the SGLT2 inhibitor can be assessed with a two-sided 95% confidence interval for the rate ratio [MA formation rate (SGLT2 inhibitor)/MA formation rate(sulfonylurea)]. From a regulatory and ethical perspective, the proposed study design is feasible, and an attempt was already done by our research group to perform such a study, which was registered on ClinicalTrials.gov (identifier: NCT02985242). Patient recruitment however, in this very specific indication in the required developmental state of DR is obviously very hard to achieve for a university hospital. In our approach we conducted a mono-center investigator-initiated trial in this indication and despite extensive efforts and time, we were unfortunately unable to recruit the statistically necessary number of patients. Some of the reasons for the unsuccessful recruitment were: marked and very rapid success and entry of SGLT2 inhibitors as standard blood sugar treatment in patients with diabetes after the proven beneficial results of cardiovascular outcome trials had been published, the study population we were looking for had no discernable visual loss and, thus, no disease burden which would encourage patients to participate in a clinical trial, low time flexibility of the target population which was still in working life, and a time-demanding study with frequent visits in the study center for the participants with low patient compensation. We suggest performing the proposed study as a multicenter trial with at least five very dedicated study centers with experience in the respective indication.

## Summary and outlooking statement on suited methodology to be used in a proof of concept trial designed to investigate efficacy of SGLT2 inhibitors in DR

Prevalence of DR is expected to rise further over the coming decades and up to now no targeted treatment is available to reduce progress in the early stages of DR. Thus, preventative therapies are urgently needed. Treatment with SGLT2 inhibitors simultaneously reduces glucotoxicity,^23^ improves insulin sensitivity and β-cell function,^22^ reduces blood pressure and body weight,^17,18^ and is therefore suggested as a potential favorable and preventative treatment option for patients with progressing DR.^26^ However, a computer-based simulation study alone, which has already been performed by Dziuba and colleagues showing overwhelming efficacy of SGLT2 inhibitors in this indication, is not sufficient to prove treatment efficacy and safety prospectively. Hard evidence is needed that can only be provided by data from a randomized controlled multicenter trial. MA formation rate is regarded as a feasible biomarker and primary study endpoint.
